# Supplementary material for: Oncogenic BRAF induces whole-genome doubling through suppression of cytokinesis
Source: Nat Commun. 2022 Jul 15;13:4109. doi: 10.1038/s41467-022-31899-9 (PMC9287415; doi:10.1038/s41467-022-31899-9)
Supplement: Supplementary file 1 — Supplementary Information [file 41467_2022_31899_MOESM1_ESM.pdf]

Supplementary Information

Oncogenic BRAF Induces Whole-Genome Doubling Through Suppression of Cytokinesis

Revati Darp[1,2], Marc A. Vittoria[3], Neil J. Ganem[3], Craig J. Ceol[1,2]

[1] University of Massachusetts Chan Medical School, Program in Molecular Medicine, Worcester, MA, USA

[2] University of Massachusetts Chan Medical School, Department of Molecular, Cellular and Cancer Biology, Worcester, MA, USA

[3] Departments of Pharmacology and Experimental Therapeutics and Medicine, Division of Hematology and Oncology, Boston University School of Medicine, Boston, MA, USA

This file contains 8 Supplementary Figures and Legends.

SUPPLEMENTARY FIGURE 1

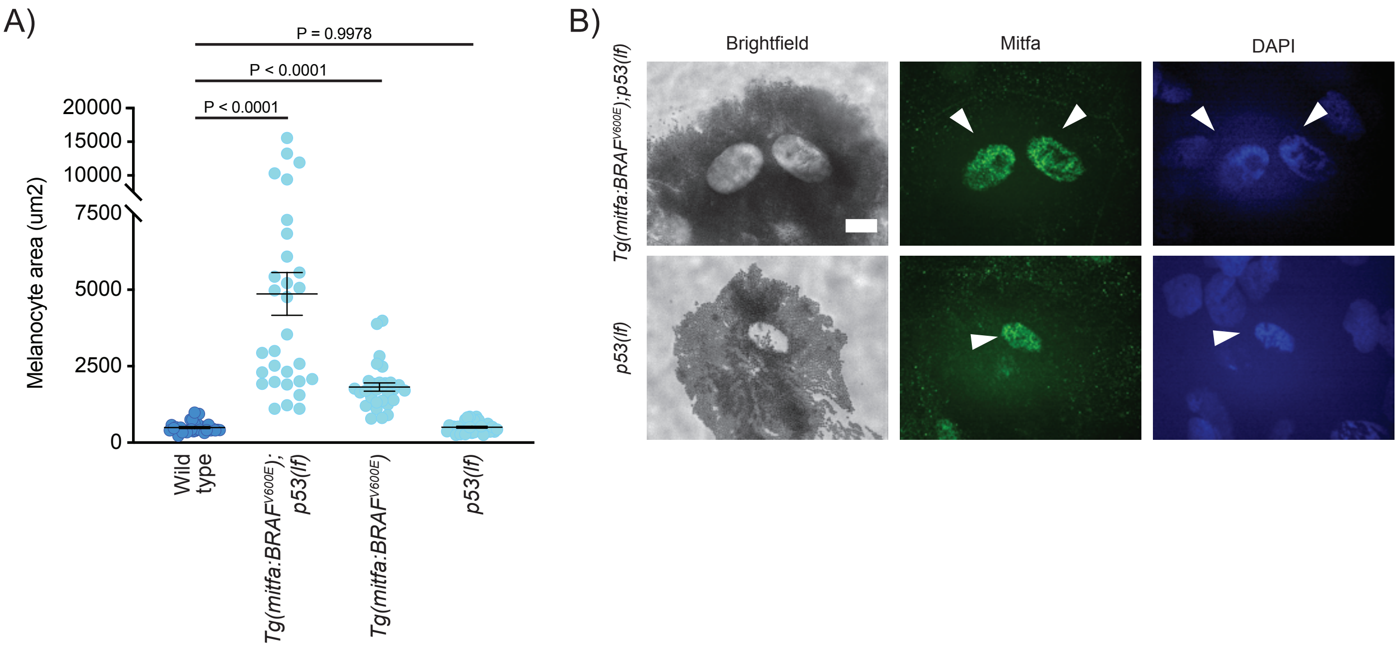

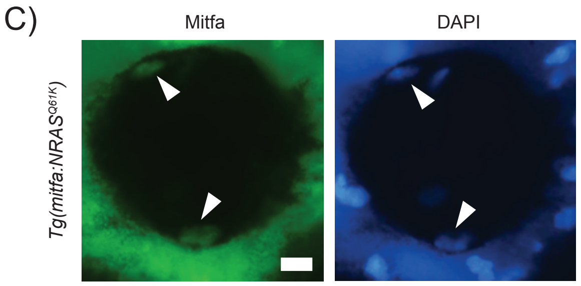

D)

	Percent mononucleate	Percent binucleate	n
control	92.9	7.1	225
BRAF^{V600E}	9.7	90.3	299

Supplementary Figure 1: Oncogenic BRAF and NRAS cause melanocytes in zebrafish to be binucleate

A) Areas of melanocytes from Wild-type, *Tg(mitfa:BRAF^{V600E});p53(lf)*, *Tg(mitfa:BRAF^{V600E})* and *p53(lf)* strains. *N* = 30 for all genotypes. Brown-Forsythe and Welch one-way ANOVA with Dunnett's multiple comparisons test. Error bars represent mean ± SEM.

B) Images from brightfield (left), anti-Mitfa (middle) and DAPI (right) staining of a single *Tg(mitfa:BRAF^{V600E}); p53(lf)* (top) or *p53(lf)* (bottom) epidermal melanocyte. Only the melanocyte nuclei stain positively for Mitfa. White arrowheads indicate nuclei within a single melanocyte. Scale bar = 5μm. Representative cells quantified in Figure 1E are shown.

C) Images from anti-Mitfa (left) and DAPI (right) staining of a single *Tg(mitfa:NRAS^{Q61K})* epidermal melanocyte. White arrowheads indicate nuclei within a single melanocyte. Scale bar = 20μm. Representative cells quantified in Figure 1E are shown.

D) Quantification of percent mononucleate and binucleate melanocytes from *Tg(mitfa:EGFP);alb(lf)* and *Tg(mitfa:EGFP); Tg(mitfa:BRAF^{V600E}); alb(lf)* strains using flow cytometry. Chi-square test, p=0.000009.

SUPPLEMENTARY FIGURE 2

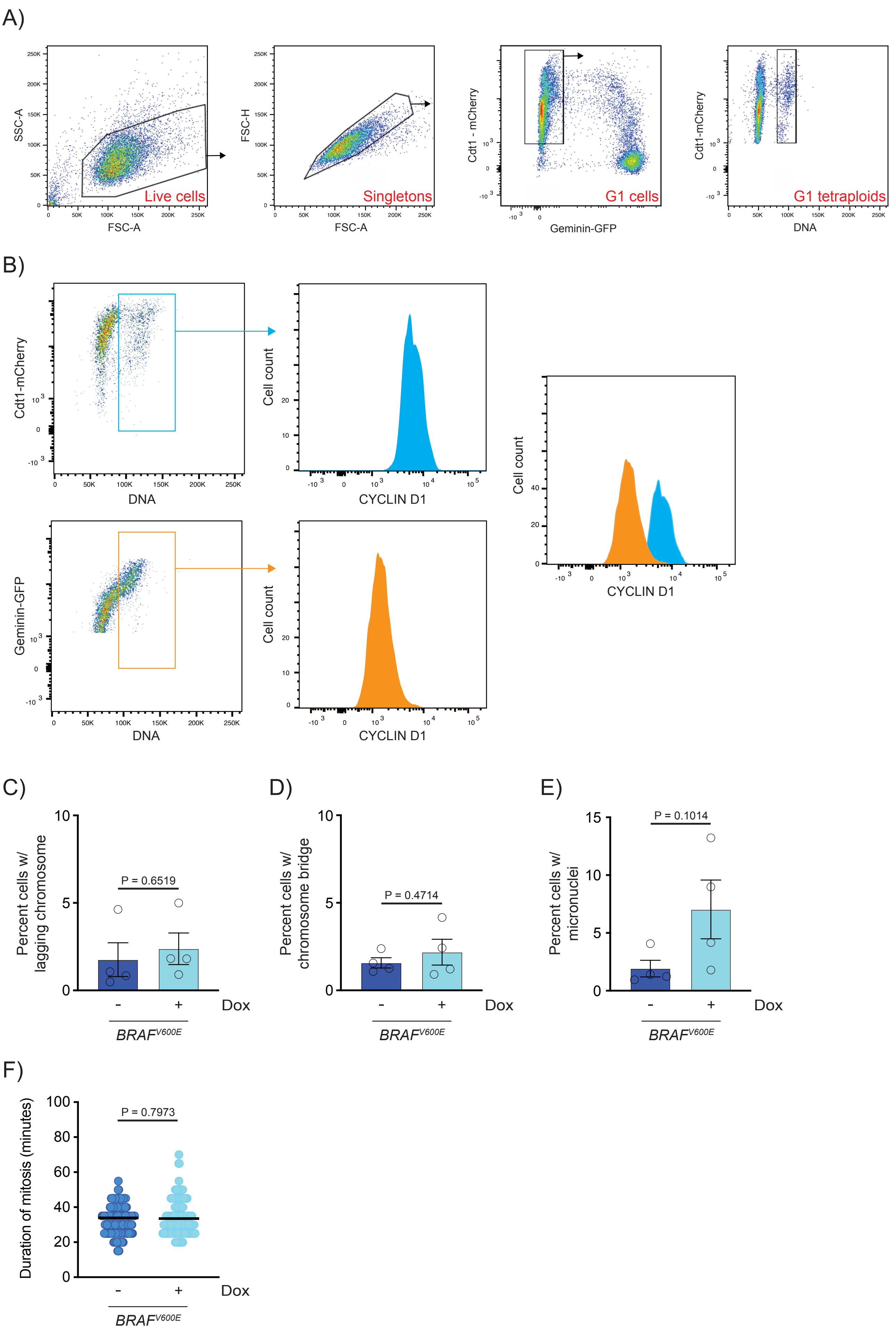

Supplementary Figure 2: Characterization of chromosomal and cell cycle features of *BRAF*^{V600E}-induced binucleate cells

A) Flow gating strategy. From left to right: SSC/FSC-A for live cell gating, FSC-H/FSC-A for single cells, Cdt1-mCherry/Geminin-GFP for G1 cells, Cdt1-mCherry/DNA for G1 tetraploids. Gates were determined to unstained/non-fluorescent negative controls.

B) Flow cytometry plots of Cyclin D1 analysis of RPE-1 FUCCI cells in G1 vs S/G2/M phases. Gating of Cdt1-mCherry-positive 4N cells (top left) and Geminin-GFP-positive 4N cells (bottom left). CYCLIN D1 staining of 4N populations (middle), and an overlay of CYCLIN D1 histograms (right) comparing Cdt1-mCherry-positive 4N cells (blue) and Geminin-GFP-positive 4N cells (orange).

C) Percent control RPE-1 cells and *BRAFV600E*-expressing RPE-1 cells with lagging chromosomes. *N* = 4 independent experiments examining -Dox = 934 and +Dox = 568 cells. Unpaired Student's *t* test. Error bars represent mean ± SEM.

D) Percent control RPE-1 cells and *BRAFV600E*-expressing RPE-1 cells with chromosome bridges. *N* = 4 independent experiments examining -Dox = 934 and +Dox = 568 cells. Unpaired Student's *t* test. Error bars represent mean ± SEM.

E) Percent control RPE-1 cells and *BRAFV600E*-expressing RPE-1 cells with micronuclei. *N* = 4 independent experiments examining -Dox = 934 and +Dox = 568 cells. Unpaired Student's *t* test. Error bars represent mean ± SEM.

F) Duration of mitosis in control RPE-1 and *BRAF*^{V600E}-expressing RPE-1 cells as quantified from live-cell imaging experiments. Each data point represents a single cell. *N* = 200 cells for -Dox and +Dox. Unpaired Student's *t* test. Mean duration is indicated.

SUPPLEMENTARY FIGURE 3

A)

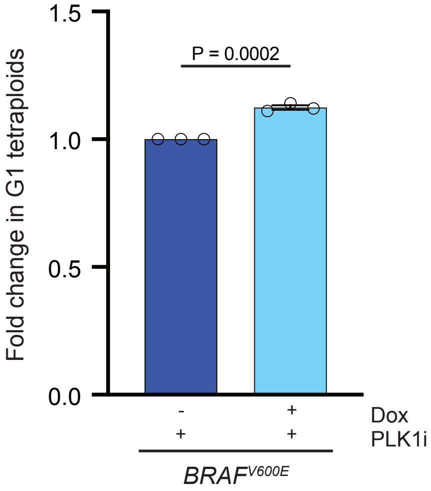

B)

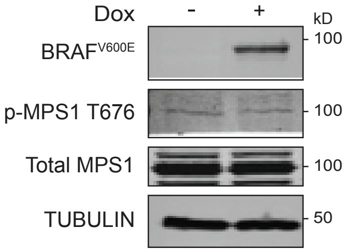

Supplementary Figure 3: Evaluation of PLK1 and MPS1 in *BRAF*^{V600E}-induced tetraploidy

A) Fold change in G1 tetraploid RPE-1 FUCCI cells following addition of the PLK1 inhibitor volasertib. Volasertib was added coincident with Dox administration. Fold change in G1 tetraploids relative to the control (+Dox no drug) are shown. *N* = 3 independent experiments. Unpaired Student's *t* test. Error bars represent mean ± SEM.

B) Western blot of phosphorylated MPS1/TTK1 and total MPS1/TTK1 in *BRAF*^{V600E}-expressing RPE-1 FUCCI cells. Synchronized control (-Dox) and *BRAF*^{V600E}-expressing (+Dox) mitotic RPE-1 FUCCI cells were harvested 12hrs post thymidine release. Tubulin was used as a loading control. A representative of three biological replicates is shown.

SUPPLEMENTARY FIGURE 4

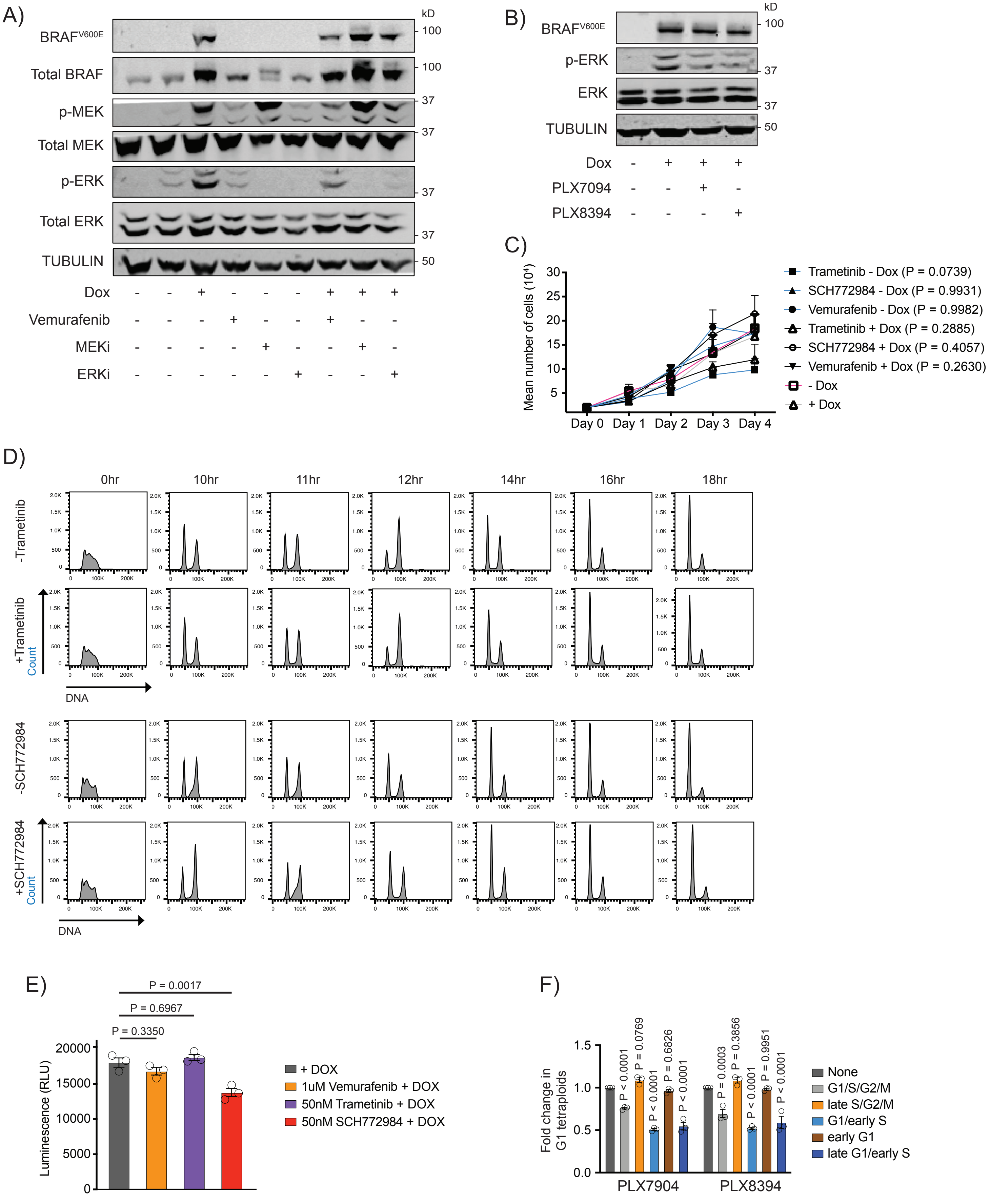

Supplementary Figure 4: Effects of RAS/MAPK inhibitory drugs on RPE-1 control cells and *$BRAF^{V600E}$*-induced tetraploidy

A) Western blot of phosphorylated MEK and phosphorylated ERK upon treatment with BRAF, MEK and ERK inhibitors. Synchronized control (-Dox) or *$BRAF^{V600E}$*-expressing (+Dox) RPE-1 FUCCI cells were harvested 48hrs following incubation with inhibitors. Inhibitors used were the BRAF inhibitor Vemurafenib, MEK inhibitor Trametinib (MEKi) and ERK inhibitor SCH772984 (ERKi). Tubulin was used as a loading control. Western blots quantified in Figure 4A are shown. A representative of four biological replicates for each blot is shown.

B) Western blot of phosphorylated ERK upon treatment with BRAF paradox-breaking inhibitors. Synchronized control (-Dox) or *$BRAF^{V600E}$*-expressing (+Dox) RPE-1 FUCCI cells were harvested 48hrs following incubation with inhibitors. Inhibitors used were the BRAF paradox-breaking inhibitors PLX7094 and PLX8394. Tubulin was used as a loading control. A representative of three biological replicates for each blot is shown.

C) Proliferation curves of control RPE-1 FUCCI cells and *BRAFV600E*-expressing RPE-1 FUCCI cells treated with Trametinib (MEKi), SCH772984 (ERKi) and Vemurafenib (BRAFi). Cells were counted every 24hrs to record cell counts. Each point represents mean number of cells from three independent experiments. Two-way ANOVA with Dunnett's multiple comparisons test, drug + Dox relative to control + Dox cells, drug – Dox relative to control -Dox cells. P values are given for comparisons at Day 4; none of the same comparisons from Days 1-3 had P values less than 0.05. Error bars represent mean ± SEM.

D) Flow cytometry plots showing cell cycle progression of control untreated RPE-1 FUCCI cells and cells treated with Trametinib (MEKi) and SCH772984 (ERKi). The 0hr timepoint corresponds to when cells were released from thymidine block. Representative plots from three independent experiments are shown.

E) Caspase-glo assay to quantify apoptosis of control *$BRAF^{V600E}$*-expressing RPE-1 FUCCI cells and *$BRAF^{V600E}$*-expressing RPE-1 FUCCI cells treated with Trametinib (MEKi), SCH772984 (ERKi) and Vemurafenib (BRAFi). *N* = 3 independent experiments. One-way ANOVA with Tukey's multiple comparisons test. Error bars represent mean ± SEM.

F) Fold change in G1 RPE-1 FUCCI tetraploids following BRAF paradox-breaking inhibitor treatment. Fold changes are expressed relative to control (+*$BRAF^{V600E}$*, no drug) samples. Inhibitors were added at indicated timepoints. *N* = 3 independent experiments. One-way

ANOVA with Dunnett's multiple comparisons test; P values are given relative to control cells. Error bars represent mean ± SEM.

SUPPLEMENTARY FIGURE 5

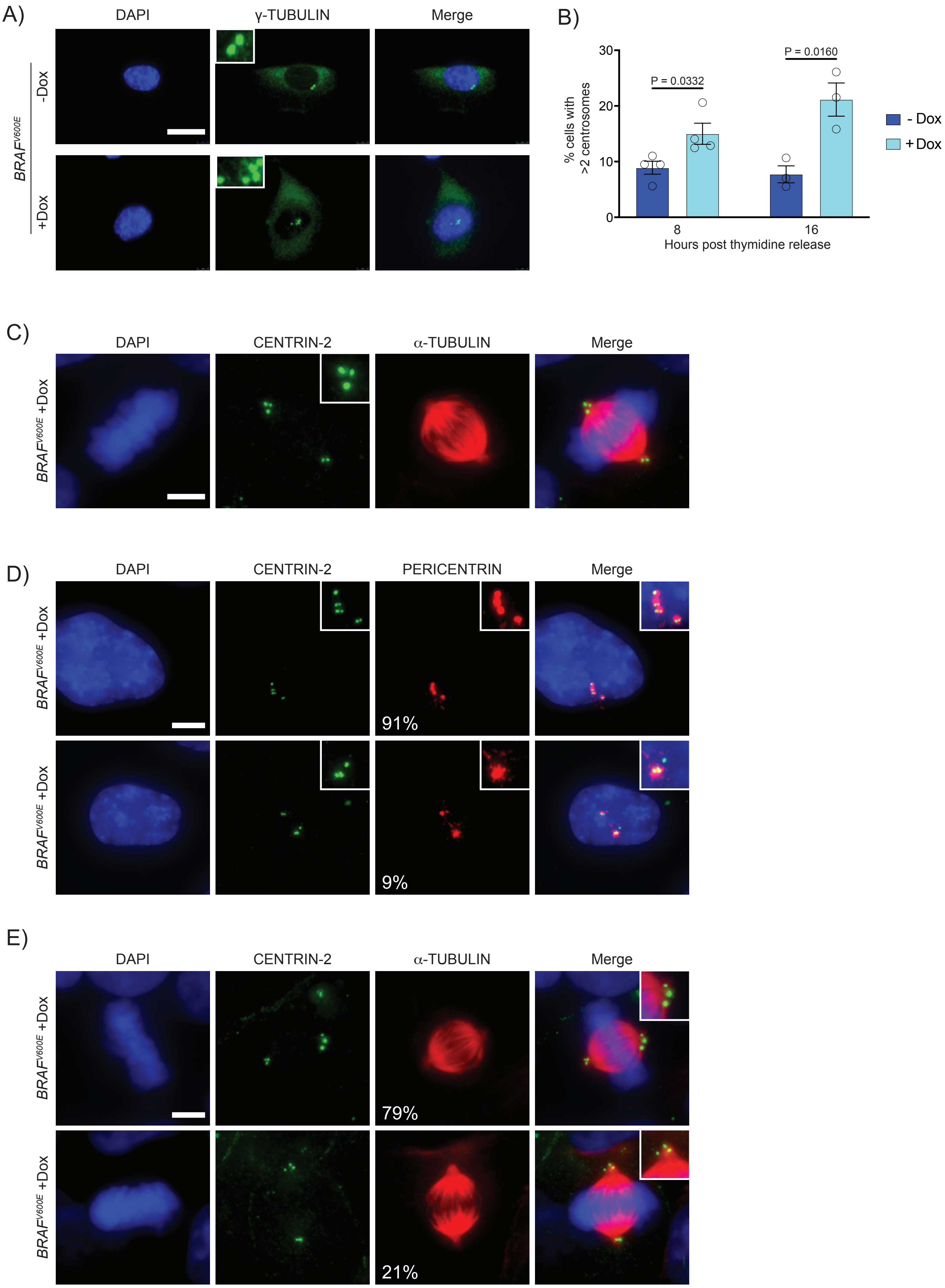

Supplementary Figure 5: Analysis of supernumerary centrioles in *BRAF^{V600E}*-expressing cells

A) DAPI and anti-γ-TUBULIN staining of control (-Dox) and *BRAF^{V600E}*-expressing (+Dox) RPE-1 cells. Images shown were taken of S/G2 cells at 8hrs post thymidine release. Insets show centrosomes. Scale bar= 7.5μM.

B) Percent control RPE-1 cells and *BRAF^{V600E}*-expressing RPE-1 cells with >2 centrosomes quantified at 8 and 16 hours post thymidine release. *N* = 4 independent experiments for 8hrs and *N* = 3 independent experiments for 16hrs, examining -Dox 8hrs = 1105, +Dox 8hrs = 1093, -Dox 16hrs = 1445, and +Dox 16hrs = 1262 cells. Unpaired Student's *t* test. Error bars represent mean ± SEM.

C) DAPI and anti-CENTRIN-2 staining of *BRAF^{V600E}*-expressing (+Dox) mitotic RPE-1 cells. Insets show centrioles at one pole. Merged image shows clustering of supernumerary centrosomes at one spindle pole. Scale bar = 7.5μM.

D) DAPI, anti-CENTRIN-2 and anti-PERICENTRIN staining of *BRAF^{V600E}*-expressing RPE-1 cells. Images shown were taken of S/G2 cells at 8hrs post thymidine release. Insets show centrioles and PERICENTRIN associated with centrioles. The percentages of cells with (top row) or without (bottom row) PERICENTRIN accumulation at three or more foci of single centrioles or centriole pairs is indicated. Scale bar = 7.5μM.

E) DAPI, anti-CENTRIN-2 and anti-α-TUBULIN staining of *BRAF^{V600E}*-expressing (+Dox) mitotic RPE-1 cells. Insets show centrioles and microtubules associated at a spindle pole. The percentages of cells with (top row) or without (bottom row) α-TUBULIN emanating from two or more foci of single centrioles or centriole pairs is indicated. Scale bar = 7.5μM.

SUPPLEMENTARY FIGURE 6

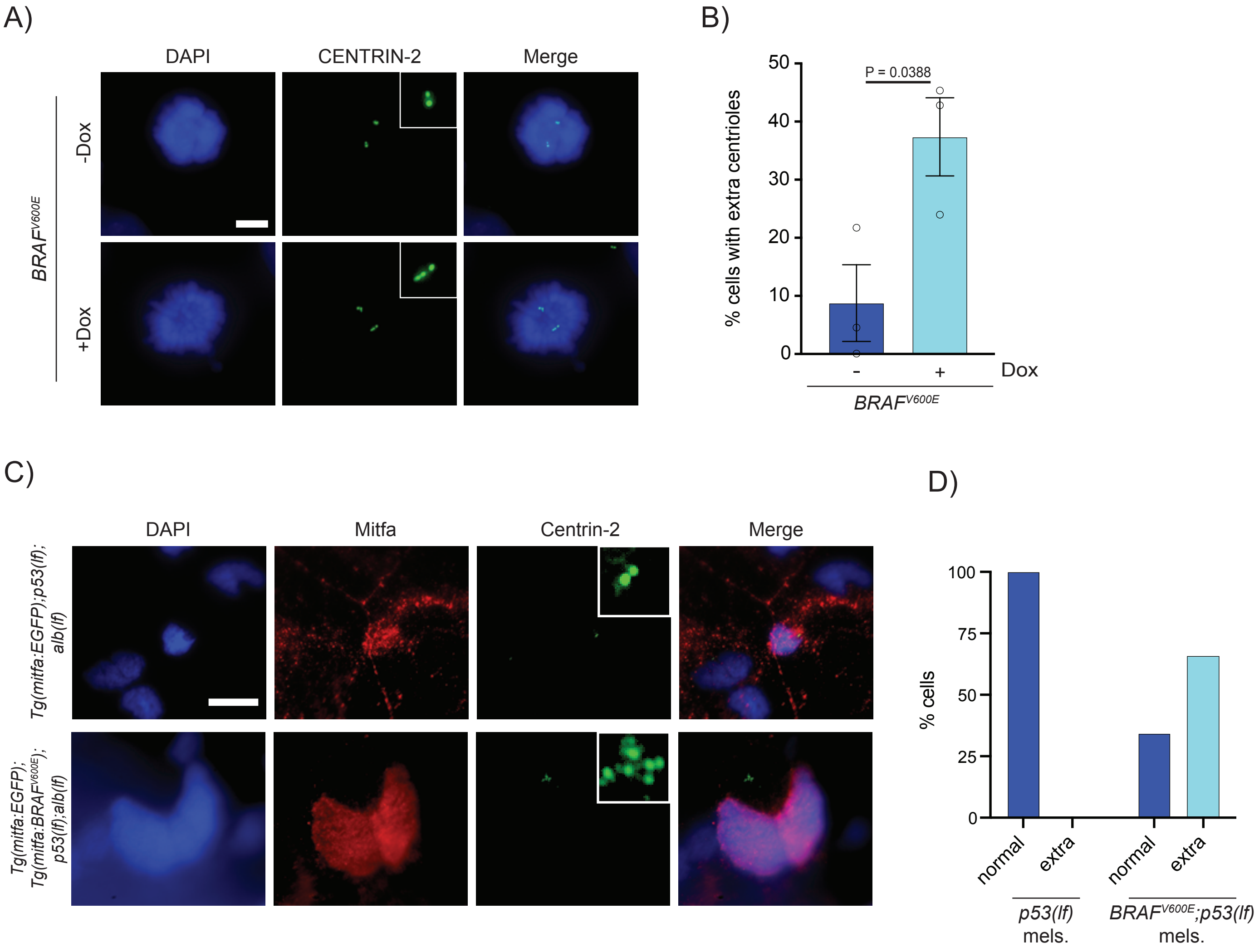

Supplementary Figure 6: Supernumerary centrioles are observed in *BRAFV600E*-expressing Mel-ST cells and zebrafish melanocytes

A) DAPI and anti-CENTRIN-2 staining in control (-Dox) and $BRAF^{V600E}$-expressing (+Dox) anaphase Mel-ST cells. Insets show centrioles at one pole. Images are maximum intensity projections of z-stacks. Scale bar = 7.5μM.

B) Percent control RPE-1 and $BRAF^{V600E}$-expressing RPE-1 mitotic cells with with supernumerary (>4) centrioles. Drugs were added coincident with Dox administration. *N* = 3 independent experiments examining -Dox =131 and +Dox =169 cells. Unpaired Student's *t* test. Error bars represent mean ± SEM.

C) DAPI, anti-Mitfa and anti-Centrin-2 staining of *Tg(mitfa:EGFP); p53(lf); alb(lf)* and *Tg(mitfa:EGFP); Tg(mitfa:*$BRAF^{V600E}$*); p53(lf); alb(lf)* in non-cycling zebrafish melanocytes. Scale bar = 7.5μM. Insets show centrioles.

D) Percent cells with normal and extra centrioles in control *Tg(mitfa:EGFP); p53(lf); alb(lf)* and *Tg(mitfa:EGFP); Tg(mitfa:*$BRAF^{V600E}$*); p53(lf); alb(lf)* zebrafish melanocytes. Normal centrioles are 2 per nucleus, so extra centrioles are >2 in mononuclear *Tg(mitfa:EGFP); p53(lf); alb(lf)* cells and >4 in binucleate *Tg(mitfa:EGFP); Tg(mitfa:*$BRAF^{V600E}$*); p53(lf); alb(lf)* cells. *N* = 38 melanocytes for *Tg(mitfa:EGFP); p53(lf); alb(lf)* and *N* = 40 for *Tg(mitfa:EGFP); Tg(mitfa:*$BRAF^{V600E}$*); p53(lf); alb(lf)*. Chi-square test p = 0.000009.

SUPPLEMENTARY FIGURE 7

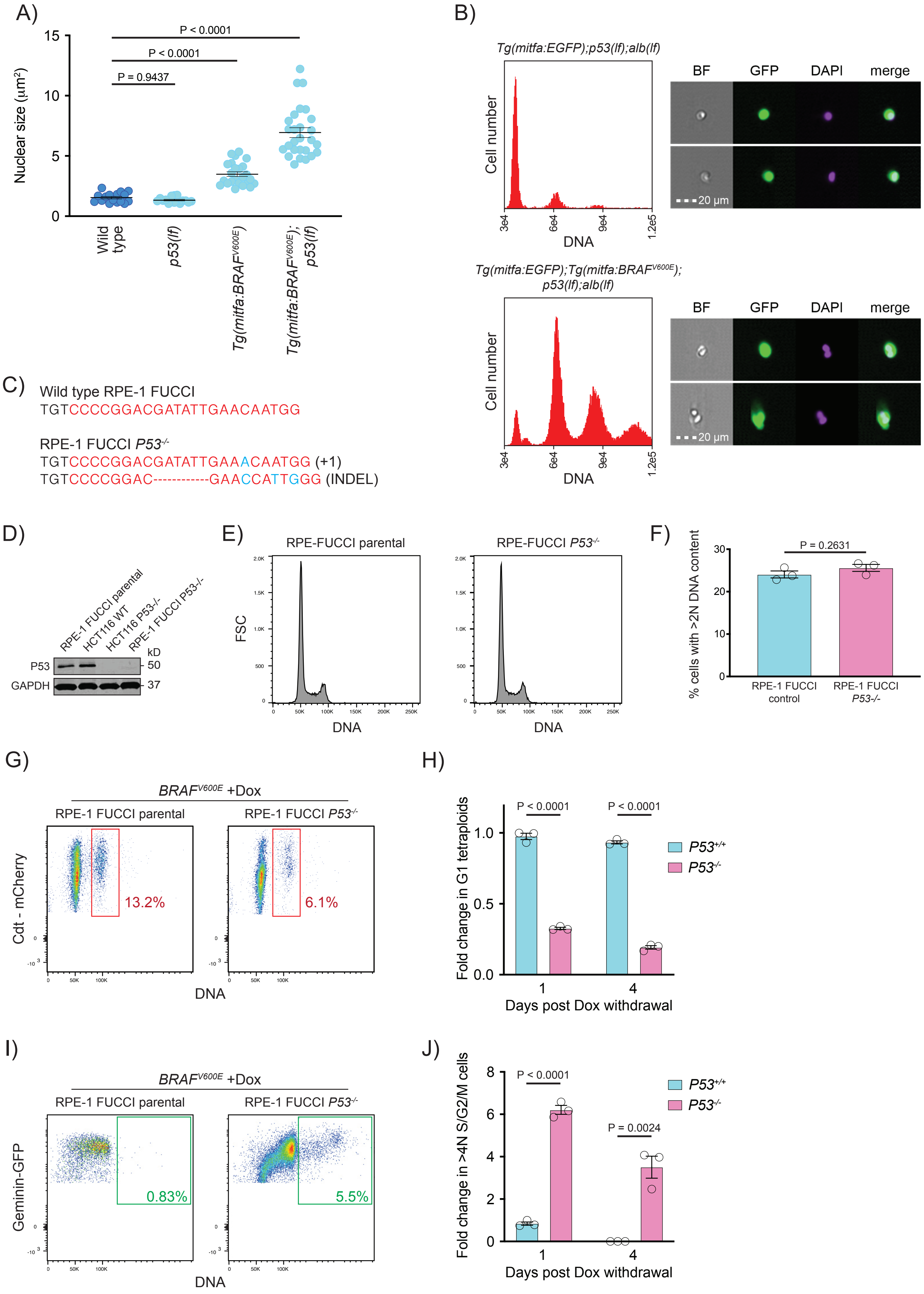

Supplementary Figure 7: Loss of *P53* enables *BRAF^{V600E}*-induced tetraploid cells to bypass a block in DNA replication

A) Nuclear sizes of Wild-type, *p53(lf)*, *Tg(mitfa:BRAF^{V600E})* and *Tg(mitfa:BRAF^{V600E}); p53(lf)* strains. In all strains *Tg(mitfa:EGFP)* and *alb(lf)* were in the strain background to enable visualization of nuclei. *N* = 16 melanocyte nuclei for Wild type, *N* = 16 for *p53(lf)*, *N* = 24 for *Tg(mitfa:BRAFV600E)*, and *N* = 27 for *Tg(mitfa:BRAF^{V600E}); p53(lf)*. One-way ANOVA with Dunnett's multiple comparisons test. Error bars represent mean ± SEM.

B) Flow cytometry and DNA content analysis of *Tg(mitfa:EGFP); p53(lf); alb(lf)* and *Tg(mitfa:EGFP); Tg(mitfa:BRAF^{V600E}); p53(lf); alb(lf)* melanocytes with brightfield, EGFP and DAPI images of single melanocytes.

C) Genotype of Crispr/Cas9-targeted RPE-1 FUCCI P53$^{-/-}$ cells.

D) Western blot of P53 in RPE-1 FUCCI P53$^{+/+}$ compared to RPE-1 FUCCI P53$^{-/-}$ cells. HCT116 P53$^{+/+}$ and HCT116 P53$^{-/-}$ cells were used as controls. GAPDH was a loading control. A representative of two biological replicates is shown.

E) Flow cytometry histograms comparing DNA content of unsynchronized RPE-1 FUCCI control cells and RPE-1 FUCCI P53$^{-/-}$ cells.

F) Percent control parental RPE-1 FUCCI cells and *P53$^{-/-}$* RPE-1 FUCCI cells with >2N DNA content. *N* = 3 independent experiments. Unpaired Student's *t* test. Error bars represent mean ± SEM.

G) Flow cytometry plots of *BRAF^{V600E}*-expressing (+Dox) RPE-1 FUCCI parental and RPE-1 FUCCI P53$^{-/-}$ cells. Tetraploid cells accumulating in G1 were quantified based on Cdt1-mCherry positivity and Hoechst incorporation. Percentages of G1 tetraploid cells in control and *BRAF^{V600E}*-expressing cultures are indicated.

H) Fold change in G1 tetraploid *BRAF^{V600E}*-expressing control parental RPE-1 FUCCI and *P53$^{-/-}$* RPE-1 FUCCI cells. Fold changes for one day and four days after Dox withdrawal are shown. *N* = 3 independent experiments. Unpaired Student's *t* test. Error bars represent mean ± SEM.

I) Flow cytometry plots of *BRAF^{V600E}*-expressing (+Dox) RPE-1 FUCCI parental and RPE-1 FUCCI P53$^{-/-}$ cells. Tetraploid cells accumulating in S/G2/M were quantified based on Geminin-GFP positivity and Hoechst incorporation. Percentages of >4N S/G2/M cells in control and *BRAF^{V600E}*-expressing cultures are indicated.

J) Fold change in >4N S/G2/M *BRAF^{V600E}*-expressing control parental RPE-1 FUCCI and *P53$^{-/-}$* RPE-1 FUCCI cells. Fold changes for one day and four days after Dox withdrawal

are shown. $N = 3$ independent experiments. Unpaired Student's t test. Error bars represent mean ± SEM.

SUPPLEMENTARY FIGURE 8

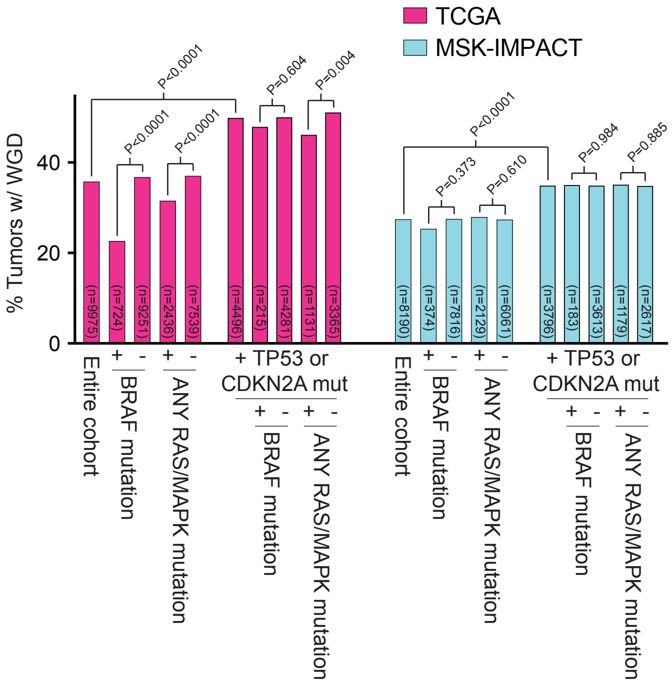

Supplementary Figure 8: Analysis of association of BRAF, any RAS/MAPK and P53/CDKN2A mutations with whole-genome doubling in human tumors

Percentages of whole-genome doubling (WGD) in human tumors with specified genotypes. The number of tumor samples is indicated in parentheses. There is a negative correlation between BRAF and ANY RAS/MAPK mutations with WGD in the TCGA cohort, but this negative correlation is not observed in the MSK-IMPACT cohort. The reason for this difference is not clear but could be related to different sensitivity and mutation calling used for the two different cohorts. As has been previously discovered [1,2], there is a strong positive correlation between mutations in TP53 and WGD, and our observations with TP53 or CDKN2A mutations reflect this correlation. As seen in the TCGA cohort, the negative correlation of BRAF and ANY RAS/MAPK mutations with WGD in the absence of TP53/CDKN2A mutations could reflect a block in cell cycle progression that occurs when BRAF or ANY RAS/MAPK activation occurs with an intact TP53 pathway. Additionally, the data suggest that a substantial fraction of BRAF or ANY RAS/MAPK mutant tumors exhibit WGD, and WGD in these tumors may be enabled by TP53 pathway loss, but BRAF or ANY RAS/MAPK mutations do not appear to alone drive WGD. P values from Chi-square exact test with Yates' correction.

References

1 Taylor, A. M. *et al.* Genomic and Functional Approaches to Understanding Cancer Aneuploidy. *Cancer Cell* **33**, 676-689 e673, doi:10.1016/j.ccell.2018.03.007 (2018).

2 Bielski, C. M. *et al.* Genome doubling shapes the evolution and prognosis of advanced cancers. *Nat Genet* **50**, 1189-1195, doi:10.1038/s41588-018-0165-1 (2018).
